# The Effects of Species Abundance, Spatial Distribution, and Phylogeny on a Plant-Ectomycorrhizal Fungal Network

**DOI:** 10.3389/fpls.2022.784778

**Published:** 2022-05-18

**Authors:** Chunchao Zhu, Zihui Wang, David C. Deane, Wenqi Luo, Yongfa Chen, Yongjun Cao, Yumiao Lin, Minhua Zhang

**Affiliations:** ^1^Department of Bioengineering, Zhuhai Campus of Zunyi Medical University, Zhuhai, China; ^2^Département des Sciences Biologiques, Université du Québec à Montréal, Montréal, QC, Canada; ^3^Centre for Future Landscapes and Department of Environment and Genetics, La Trobe University, Bundoora, VIC, Australia; ^4^Department of Ecology, Sun Yat-sen University, Guangzhou, China; ^5^ECNU-Alberta Joint Lab for Biodiversity Study, Zhejiang Tiantong National Station for Forest Ecosystems, East China Normal University, Shanghai, China

**Keywords:** plant-fungus interaction, ectomycorrhizal fungus, network structure, community assembly, roots, symbiotic network

## Abstract

Plant and root fungal interactions are among the most important belowground ecological interactions, however, the mechanisms underlying pairwise interactions and network patterns of rhizosphere fungi and host plants remain unknown. We tested whether neutral process or spatial constraints individually or jointly best explained quantitative plant–ectomycorrhizal fungal network assembly in a subtropical forest in southern China. Results showed that the observed plant–ectomycorrhizal fungal network had low connectivity, high interaction evenness, and an intermediate level of specialization, with nestedness and modularity both greater than random expectation. Incorporating information on the relative abundance and spatial overlap of plants and fungi well predicted network nestedness and connectance, but not necessarily explained other network metrics such as specificity. Spatial overlap better predicted pairwise species interactions of plants and ectomycorrhizal fungi than species abundance or a combination of species abundance and spatial overlap. There was a significant phylogenetic signal on species degree and interaction strength for ectomycorrhizal fungal but not for plant species. Our study suggests that neutral processes (species abundance matching) and niche/dispersal-related processes (implied by spatial overlap and phylogeny) jointly drive the shaping of a plant-ectomycorrhizal fungal network.

## Introduction

The plant–ectomycorrhizal (EM) fungal network is one of the most common mutualistic symbiotic networks known. Both plants and their EM fungal partners are obligate symbionts, as they cannot complete their life cycle without forming a symbiosis (Taylor, 2008). The selection and adaptation of ectomycorrhizal fungi (among the most diverse mycorrhizal fungi) to their host plant roots are products of long-term ecological and evolutionary processes. This is expected to lead to specificity in the interaction between these symbiotic partners. Despite this, EM fungi are reported to have a relatively low degree of host specialization (Roy-Bolduc et al., 2016). EM fungi provide crucial benefits for plants by enhancing soil nutrient acquisition, plant seedling establishment, and disease resistance (van der Heijden et al., 2008; Liang et al., 2020; Cahanovitc and Angel, 2022) and can promote monodominance in forest communities (McGuire, 2007). In turn, host plants support their EM fungi through provision of photosynthetically fixed carbon (Högberg and Högberg, 2002). Thus, elucidating the symbiotic network patterns and factors that regulate EM fungi and host plants is important for understanding community assembly and ecosystem functioning.

Identifying the factors that drive the organization of plants and EM fungi into complex networks remains poorly explored, largely due to historical limitations in sampling and taxonomic identification of root fungi. Increasingly, ecologists are constructing high taxonomic resolution networks of plant-fungal communities using high throughput sequencing technology (Bahram et al., 2014; Chen et al., 2019; Wang et al., 2019), which have started to illustrate the network structures of EM fungi, arbuscular mycorrhizal fungi and their host plants (Dickie et al., 2017; Põlme et al., 2018; Arraiano-Castilho et al., 2020).

Two common network structures of interest include nestedness and modularity. Nestedness describes the tendency of specialized species to interact with a subset of the interaction partners of more generalized species (Bascompte et al., 2003). Modularity describes how a complex network can be organized into distinct modules, where interactions are more common within modules than between modules (Olesen et al., 2007). However, observed structures of plant-EM fungal networks vary widely, exhibiting either non-nested or anti-nested patterns (Bahram et al., 2014; Arraiano-Castilho et al., 2020) and non-modular (Bahram et al., 2014) or modular structures (Dickie et al., 2017; Arraiano-Castilho et al., 2020). The reasons for such inconsistent network structural properties remain unclear, but it is notable that most prior work used to construct plant–EM fungal networks have typically used less than a dozen species (Bahram et al., 2014; Dickie et al., 2017; Arraiano-Castilho et al., 2020). This low number of host plant species might have contributed to the failure to detect network structural properties such as nestedness or modularity. A comprehensive understanding of the community assembly rules that structure plant–EM fungal networks might then require networks that incorporate increased sampling effort for host plant species.

Species interaction patterns and structural properties of plant–EM fungal networks are also affected by ecological and evolutionary factors. A neutral view of assembly considers interactions among individuals are essentially random and it is relative species abundances that determine network patterns (Canard et al., 2012). However, deterministic factors such as the mismatch of environmental tolerances of plants and fungi (Arraiano-Castilho et al., 2021) and human effects (Wall et al., 2020) may regulate the establishment of interaction relationships by imposing spatial constraints on encounter probabilities. For instance, structures of aboveground networks are best predicted by a combination of species abundance and other deterministic factors such as spatial overlap (Vázquez et al., 2009; Sáyago et al., 2013), phylogeny (Cagnolo et al., 2011) and trait matching (Olito and Fox, 2015). In belowground networks, spatial overlap of plant and fungus communities is also expected to regulate the structure of plant-root fungal networks. However, a lack of substantial structure in the co-occurrence network of plants and arbuscular mycorrhizal fungi (Encinas-Viso et al., 2016) suggests that spatial information might have limited influence on plant-fungal network assembly. Evolutionary history must also be considered, for example, being associated with nonrandom patterns in woody plant–arbuscular mycorrhizal fungal networks (Chen et al., 2017). In general, while species abundances, spatial proximity and phylogeny all contribute to interaction patterns of plant–EM fungal and network structures, their interaction and relative contributions under different environmental conditions remain poorly understood.

To fill the gaps in understanding the processes that assemble EM fungi and host plants into complex networks, we analyzed a large interaction database constructed by next-generation sequencing involving 43 plants and 862 EM fungi in a 50 ha subtropical forest plot in southern China (Wang et al., 2019). The database is notable for the high number of host plant species, which we hypothesized might afford a more detailed understanding of the rules determining network assembly. In analyzing the database, we posed the following three questions:

1. What are the structural properties of the plant-EM fungal association network?
2. How do species abundance and spatial proximity contribute to the pairwise interaction patterns and structural properties of the plant-EM fungal network?
3. Are symbiotic patterns of plants and EM fungi conserved in plant and EM fungal phylogenies?

We tested whether observed network structures were associated with neural processes, niche/dispersal related processes or the combined effects of both. We anticipated that neutral processes might dominate network structure because environmental and spatial patterns explain little of the variance in EM fungal diversity in the study site (Wang et al., 2019). We also expected to find a strong phylogenetic signal in the network due to the dominant role of host phylogeny in explaining the diversity of EM fungi in the forest (Wang et al., 2019).

## Materials and Methods

### Sampling Site and Molecular Identification

This study was conducted in a 50-ha subtropical forest plot situated in the Heishiding nature reserve of southern China, which is located on the tropic of Cancer at 23°25′ ~ 23°29′N, 111°49′ ~ 111°55′E. Mean annual temperature and precipitation are 19.7°C and 1750 mm, respectively. The total area of the nature reserve is 4,200 ha, divided into core and experimental areas. The 50-ha forest plot was established within the 1,660 ha experimental area in 2012. All trees with a diameter at breast height (DBH) ≥ 1 cm were identified to species. In total, the plot includes about 269,000 stems of 213 woody plant species.

We analyzed an existing database of the plant–EM fungal network for the forest plot. Details regarding sampling design and taxonomic identification of root fungi (based on ITS) have been previously described (Wang et al., 2019), so we provide only a summary here. Within the 50-ha stem-mapped plot, EM fungi were quantified from 512 root samples of 43 plant species, which were collected randomly in the plot (Supplementary Figure S1). The 43 species were selected based on the phylogeny and their abundance. The 43 species included 3 species from each of *Litsea* and *Lithocarpus* (two of the most abundant genera in the plot) and 37 species from a larger set of genera where 6 species were from the same families as *Litsea* (*Lithocarpus*) and the others were from different families (Wang et al., 2019). 5–15 individuals for each plant species were randomly chosen for fine root sampling with at least three replicates taken from each individual by root tracing along different directions and pooled to generate a single sample. Root identity validation based on *rbcLa* fragment showed that 97% of fine-root samples of host plants were correctly traced. Root-associated fungi were identified by the internal transcribed spacer (ITS) region of fungal rDNA (details in Wang et al., 2019). The operational taxonomic units (OTUs) of root fungi were discriminated using a threshold of 97% sequence identity. EM fungi were identified based on a database of EM fungal taxa and lineages (Tedersoo and Smith, 2013). Our final database was a single quantitative interaction matrix of 862 EM fungal OTUs and 43 plant species. To account for the sampling inequality, each cell in each network matrix was filled with the mean of abundance (sequenced reads) of each OTU (species) of EM fungi on each host tree, and the number was rounded to the nearest integer.

### Estimating Structural Metrics of Plant—Fungal Association Network

We measured six metrics frequently used in the analysis of ecological networks: connectance, interaction evenness, interaction asymmetry (for EM fungi), nestedness, specialization and modularity. Connectance is the proportion of realized interspecific links, calculated as C=L/IJ , where *L* is the number of nonzero cells in the binary interaction network and *I* and *J* are the numbers of plant and fungus species in the network. Interaction evenness was defined as Shannon’s index (Tylianakis et al., 2007), $H=p_{ij}\log_{2}p_{ij}/\log_{2}F$ , where *F* is the total number of interactions in the whole network and $p_{ij}$ is the proportion of interactions linking plant species *i* and fungal OTU *j*. Interaction asymmetry for a given species *i* was calculated as $A_{i}=\underset{j}{\sum }d_{ij}/k_{i}$ , where *k_i_* is the number of species interacting with *i* and *d_ij_* is a measure of the symmetry of the pairwise interaction strength between *i* and *j* (Vázquez et al., 2007). Network specialization was calculated following Shannon entropy, reflecting degree of complementarity specialization of a whole plant-EM fungal network (Blüthgen et al., 2006).

We estimated the modularity of the plant–EM fungal association network using the DIRTLPAwb+ algorithm set to steps = 10^6^ searches (Beckett, 2016) in R-package bipartite (Dormann et al., 2009). Network modularity (M) ranges from 0 (low modularity) to 1 (high modularity). Nestedness was assessed using the weighted nested overlap and decreasing fill (WNODF; Almeida-Neto et al., 2008) in the R-package bipartite (Dormann et al., 2009). Network nestedness ranges from 0 (low nestedness) to 100 (perfect nestedness). To determine the significance of modularity and nestedness in observed network, we compared the observed network metric to those calculated from 1,000 null networks generated with the “swap” method (Artzy-randrup and Stone, 2005; Dormann et al., 2009). This non-sequential null model algorithm was selected because it maintains both marginal totals and connectance (number of links) in the null model networks consistent with the observed networks.

### Abundance, Spatial Overlap and Calculation of Interaction Probabilities

The abundance of each plant species was quantified as total number of individuals in the 50-ha forest plot. The abundance of each mycorrhizal fungal species (OTU) was calculated as the sum of the abundance of each fungal species (OTU) on each host tree (Wang et al., 2019), which was evaluated after subsampling each sample to 3,000 sequence reads to eliminate the effects of sample size (Wang et al., 2019). To quantify spatial overlap of plant and EM fungal species at the local scale, we compiled matrices of occurrence of each. We recorded the presence or absence of each species in each cell of a 20 × 20 m grid (hereafter quadrat) in the 50 ha plot. We thus obtained one spatial occurrence matrix for host plants and one for EM fungi, with species in rows and quadrat in the columns, and cells populated with ones for presences and zeros for absences.

We calculated interaction probability matrices to test whether network metrics were associated with neutral processes (e.g., relative species abundance) and niche/dispersal related processes (as implied by spatial overlap: presence of both species in a 20 × 20 m quadrat, which could be due to dispersal limitation or autocorrelated environmental conditions) or *via* the combined effect of both (mixed). To test the hypothesis that neutral processes dominated interactions we calculated a probability matrix (**Ab**) quantifying the relative interaction potential between a plant and a fungal species as the product of their relative abundances (Vázquez et al., 2009). To obtain interaction probability matrix expected from spatial processes (niche or dispersal based), we normalized the spatial overlap matrices **Sp** so that their elements sum to one (Vázquez et al., 2009). Thus, the probability of interaction increased with increasing spatial overlap of two species pairs, while those with no spatial overlap had zero probability of interaction. To model the combined effect of neutral and niche/dispersal related processes we calculated combined probabilities (**AbSp**) as the element-wise multiplication of matrices **Ab** and **Sp**, again normalizing the resulting matrices (Vázquez et al., 2009). These combined matrices represent the expected probability under the joint influence of species abundance and spatial overlap (analogous to an interaction term in regression modeling). Thus, we had three probability matrices quantifying relative abundance, spatial overlap and their combined effect: **Ab**, **Sp** and **AbSp**. As a null model, we also defined an equiprobable probability matrix (**Null**). In this matrix, all pairwise interactions had the same probability of occurrence, where each element was calculated as the inverse of the product of the numbers of plant and EM fungal species in the network (Vázquez et al., 2009). To evaluate whether these interaction probabilities inferred from species abundance, spatial overlap and null model matched the frequencies of interaction in observed networks, we also constructed an observed probability matrix (**Obs**), where pairwise interaction probabilities were calculated as the ratio of each element and the sum of all elements in the realized network matrix.

### Predicting Pairwise Species Interactions and Plant-EM Fungal Network Structural Properties

To evaluate the ability of interaction probability matrices to predict the network metrics (connectance, evenness, specialization, modularity, nestedness and EM fungal interaction asymmetry) we used a randomization algorithm (Vázquez et al., 2009). The algorithm assigned the total number of interactions originally observed in the network according to the three probability matrices, with the only constraint being that at least one interaction was assigned for each species (Vázquez et al., 2009).

To test the relative support for each hypothesis in explaining the pairwise interaction frequencies in the observed plant–EM fungal network (Vázquez et al., 2009), we used a maximum likelihood approach. The likelihood that a probability matrix explained the observed matrix was calculated assuming that the pairwise probability of interaction between a plant and an EM fungus followed a multinomial distribution (Vázquez et al., 2009). This likelihood was calculated using function *dmultinom* in the R-package stats. The comparisons between models were conducted using differential Akaike’s information criterion ( Δ AIC; Burnham and Anderson, 2004). Δ AIC was the difference in AIC for a probability matrix (**Ab**, **Sp**, **AbSp** and **Null**) with that for the best fitting model. The model with the lowest Δ AIC indicated the model providing the best fit to the observed data (Burnham and Anderson, 2004). In addition, to provide an effective way to scale and interpret the Δ AIC, we calculated the Akaike weights (w_i_; Burnham and Anderson, 2004). The w_i_ of each model was calculated as


$$\exp\left(-\frac{1}{2}\Delta \mathrm{AIC}\right)/\overset{R}{\underset{i=1}{\sum }}\exp\left(-\frac{1}{2}\Delta \mathrm{AIC}\right)$$

where *R* was the number of all models. The value of w_i_ ranges between 0 and 1, and the sum of all model weights is 1. The greater the weight of a model, the greater the support for that model as the best candidate model in the model set.

### Evaluating Phylogenetic Signal in Species Associations

We evaluated the influence of evolutionary history on network patterns using a phylogenetic tree of plant species in a local forest plot reconstructed by four general plant DNA barcodes (*rbcLa*, *matK*, *trnL* and ITS2; Supplementary Figure S2) and a phylogenetic tree of EM fungal species (Supplementary Figure S3) reconstructed using ITS sequences (details for the phylogenetic reconstruction are given in Supplementary Method S1). We evaluated the impact of phylogenetic history on the assemblage of interacting partners of the species quantified using the Bray–Curtis dissimilarity index and using Mantel tests to compare the correlation between the phylogenetic distance matrix of plant/EM fungi and the community similarity matrix of their interacting partners.

We tested the phylogenetic signal on species degree (number of links for each species) and species strength (the importance of a species to its partner’s set taking into account the relative abundance of the species on each partner; Barrat et al., 2004). Two of the most widely used statistics, Pagel’s *λ* (Pagel, 1999) and Blomberg’s *K* (Blomberg et al., 2003), were conducted to test the phylogenetic signal in the two network indices. The two statistical tests were implemented within the *phylosig* function in the phytools R-package (Revell, 2012). Both indices assumed the classic Brownian motion (BM) evolutionary model, and their values vary from 0 to 1 for *λ* and from 0 to > > 1 for *K*. In both cases, low values (close to 0) indicate the absence of phylogenetic signal (i.e., the trait has evolved independently of phylogeny); high values (close to 1) indicate trait evolution according to BM; and, in the case of *K*, values >1 suggest that close relatives are more similar than expected under BM. The statistical significance of *K* was assessed based on a comparison of the observed phylogenetically independent contrasts and the expected contrast under 999 randomizations (Blomberg et al., 2003), whereas the statistical significance of λ was assessed based on a comparison of the likelihood of a model accounting for the observed λ with the likelihood of a model that assumes complete phylogenetic independence (Pagel, 1999).

## Results

### Observed Plant—EM Fungal Network Properties

**Figure 1:**
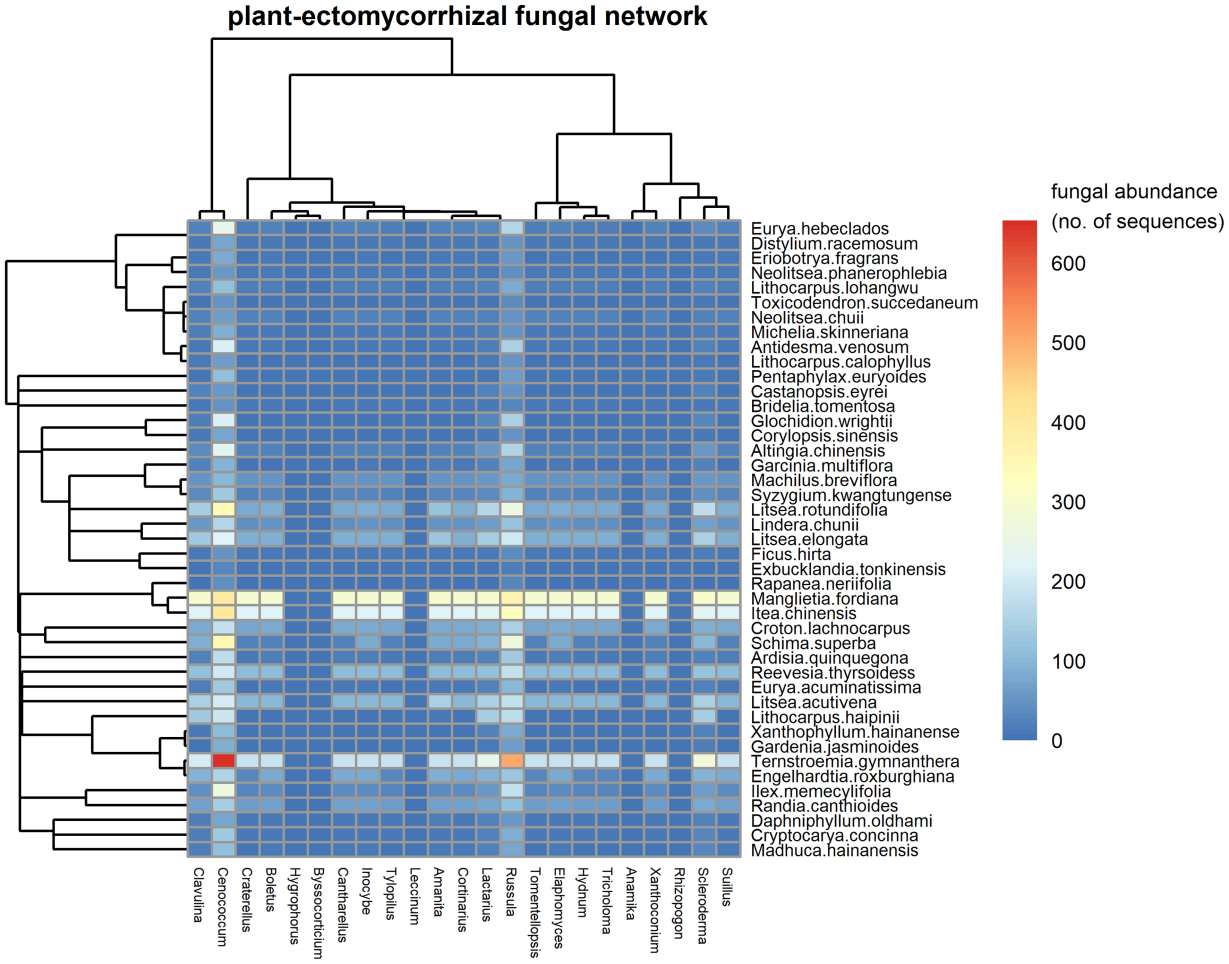
A visual plot in the plant-ectomycorrhizal (EM) fungal network. Fungal genera are shown in columns and plant species are denoted in rows. Interaction frequency of plant species and fungal genera is filled in each cell and calculated as the mean of abundance (sequenced reads) of each genus of EM fungi on each host tree with the number rounded to the nearest integer. The abundance of each genus of EM fungi on each host tree is estimated after subsampling each sample to 3,000 sequence reads. In the figure, the color scales with interaction frequencies (fungal abundances on host plants). High fungal abundances on plants are shown in red, but low fungal abundances on plants are shown in blue. The dendrograms tree for plant species (left) and EM fungal genera (top) are also shown.

The observed fungal network registered 4,360 links between the 862 EM fungal species (23 genera) and 43 woody plant species. We found that *Cenococcum* spp. and *Russula* spp. were the most important EM species in terms of their co-occurrences with roots of a high number of plant species (Figure 1). *Cenococcum* spp. and *Russula* spp. were each symbiont on 43 host plant species. Among plants, *Ternstroemia gymnanthera*, *Itea chinensis* and *Manglietia fordiana* were the species with the most interactions, associating with the roots from 23, 18 and 18 EM genera (Figure 1, 193, 147 and 92 species included), respectively. The observed plant–EM fungal network had significant strong nestedness [NODF = 10.256, CI for null model = (8.240, 8.561), *p* < 0.01]. For instance, most EM species habited on the root systems of a subset of the host plants of *Cenococcum spp*. and *Russula spp*. (Supplementary Figure S4), while most plant species established co-occurrence associations with a subset of the EM fungal symbionts of *Ternstroemia gymnanthera*, *Itea chinensis* and *Manglietia fordiana* (Supplementary Figure S4). The observed plant-EM fungal network exhibited significantly strong modular structure [M = 0.482, CI for null model = (0.058, 0.061), *p* < 0.01], with 14 modules identified (Supplementary Figure S5). The network was characterized by an intermediate level of specialization (H2′ = 0.42), low connectance (0.12) and high interaction evenness (0.74), indicating structure was not dominated by a small number of interactions. The average strength of EM fungi with their host plants was close to symmetry (mean asymmetry for EM = 0.17).

### The Role of Species Abundance and Spatial Overlap in Species Interactions and Network Structural Properties

**Figure 2:**
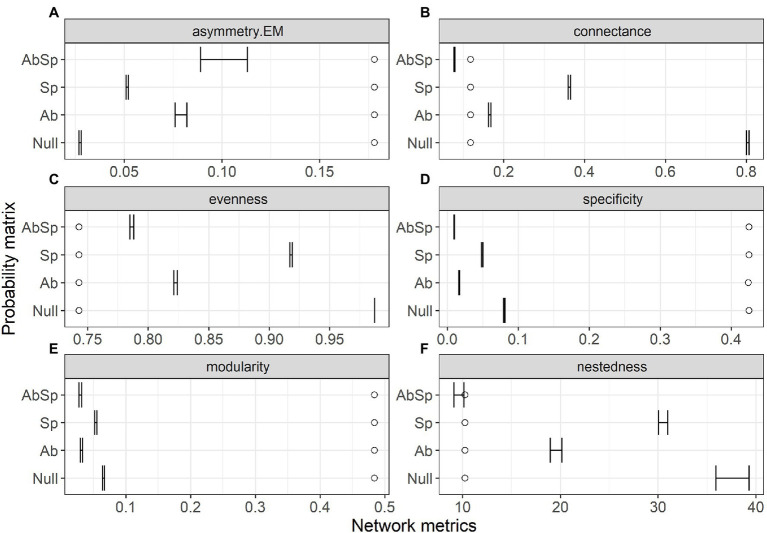
Comparison of the network metrics produced by probability matrices (95% confidence intervals) and the observed network values for the plant–ectomycorrhizal fungal network. Network metrics include interaction asymmetry for ectomycorrhizal fungus (asymmetry. EM, **A**), network connectance **(B)**, network evenness **(C)**, network specificity **(D)**, network modularity **(E)** and network nestedness **(F)**. Network metrics are shown at the top of each panel, with circles showing the observed value and lines showing the 95% confidence intervals in the predicted values. Results are shown for the three probability matrices calculated from abundance (Ab), spatial overlap (Sp), and their interaction (AbSp) and a null matrix with homogeneous interaction probabilities across all pairwise interactions (Null).

The top-ranked model for predicting species interaction frequencies was clearly that including the only spatial overlap of species (lowest Δ AIC and highest *w*AIC for the **Sp** model, Table 1) with all other models (**Ab, AbSp and null)** exceeding this by more than 100 AIC (Table 1). However, model performance differed among the network structural metrics analyzed (Figure 2). The **AbSp** model including both spatial overlap and species abundance performed best overall because the 95% confidence intervals of four out of six metrics (connectance, nestedness, evenness and asymmetry of EM) predicted by the **Absp** were more close to their observed values (Figures 2A–C,F), particularly for nestedness (Figure 2F). The **AbSp** model also provided a reasonable prediction for network connectance and was marginally better than the pure abundance model (Figure 2B). Although the **AbSp** model provided the closest prediction for interaction asymmetry for EM fungi (Figure 2A) and network evenness (Figure 2C), it was poor in absolute terms. None of the models for network specificity and modularity was an improvement on the null model (Figures 2D, E).

**Table 1:** Model selection table for predicting species interaction frequencies in the observed networks.

Rank	Model	AIC	ΔAIC	*W*_i_
1	Sp	2.98e^5^	0	1.00
2	Ab	3.32e^5^	0.34e^5^	<0.001
3	AbSp	4.20e^5^	1.22e^5^	<0.001
4	Null	5.32e^5^	2.33e^5^	<0.001

*A model with lower differential Akaike’s information criterion* ( Δ *AIC) and high Akaike weights (w_i_) is considered to have more support as the best model in the candidate set. Sp: spatial information; Ab: abundance; AbSp: the interaction of abundance and spatial information. Null: an equiprobable probability matrix*.

### Evaluating Phylogenetic Signal in Species Associations

Phylogenetic distances of plant species were significantly negatively associated with ecological similarity of the fungal community (one-tailed Mantel test *r* = −0.086, *p* = 0.025) and phylogenetic distances of fungal species were also significantly negatively related to ecological similarity of the plant community (one-tailed Mantel test *r* = −0.023, *p* = 0.001). We found no evidence of a phylogenetic signal on species degree or species strength for plants (Table 2), while for EM fungi, results were metric-specific; Pagel’s *λ s*uggested a significant phylogenetic signal in both degree and species strength (both *p* < 0.001) but this was not evident using Blomberg’s *K* (both *p* > 0.4, Table 2).

**Table 2:** Phylogenetic signal of plant-ectomycorrhizal fungal (EM) network.

Taxa	Metrics	*K* statistics	Values of *p*	λ statistics	Values of *p*
EM fungi	Degree	0.000	0.832	0.061	<0.001
	Species strength	0.000	0.402	0.938	<0.001
Host plants	Degree	0.039	0.094	0.000	1.000
	Species strength	0.000	0.851	0.000	1.000

**K* and *λ* statistics indicate the strength of the phylogenetic signal in the degree and specie strength. Value of *p* denotes the probability that degree and species strength deviate from the expectations of a Brownian motion evolution model*.

## Discussion

### Network Structure

The local plant–EM fungal network in this subtropical forest was characterized by higher species number and more interactions than comparable studies (Jacquemyn et al., 2011; Montesinos-Navarro et al., 2012; Toju et al., 2014). Consistent with other mutualistic plant-fungal networks (Jacquemyn et al., 2011; Montesinos-Navarro et al., 2012; Toju et al., 2014), our network had low connectivity (Montesinos-Navarro et al., 2012). Compared with low levels of specialization (Roy-Bolduc et al., 2016), more intermediate levels of specialization as observed here (H2′= 0.42) in the plant-EM fungal network may be advantageous for host trees by increasing their chance to form a symbiosis with suitable EM fungal partners. Nonrandom associations of plants with symbiotic partners are common in biotrophic fungi, which may enhance modular structure in our plant-EM fungal network (Supplementary Figure S5). This is an important mechanism that leads to niche partitioning (reflecting ecological specialization; Jacquemyn et al., 2015). This implies that modularity and specialization are possible mechanisms allowing many EM fungal species to coexist locally on the same set of host plants.

We found that nested structures in the plant-EM fungi network where host plants of specialized EM fungi were subsets of the host plants of generalized *Cenococcum* spp. and *Russula* spp. fungi (Supplementary Figure S4). In contrast, anti-nested structures, in which co-occurring plant species share fewer fungal symbionts than expected by chance, have been reported in many smaller plant-EM fungal networks (Bahram et al., 2014; Roy-Bolduc et al., 2016; Arraiano-Castilho et al., 2020). This suggests that increasing numbers of species might favor greater network nestedness, as suggested by Bascompte et al. (2003). As the sampling effort invested in our study over a 50-ha forest extent exceeds many other studies in plant–root fungal association networks (Jacquemyn et al., 2011; Montesinos-Navarro et al., 2012; Toju et al., 2014), we believe the sampling effort is likely to have weak if any, influence on our estimates of network metrics (see Supplementary Figure S6).

### Determinants of Pairwise Interactions in a Network

Spatial co-occurrence patterns of plant and fungal species determined species interaction frequencies while species abundance or the interaction of the two had no support in our study (Table 1). Some evidence suggests that tropical mycorrhizal populations show significant spatial heterogeneity and nonrandom associations with different hosts (Husband et al., 2002). This clarifies how species interactions may be mainly determined by plant partner choice for EM fungi, which is also controlled by soil environment heterogeneity or spatial proximity (Cavender-Bares et al., 2009; Wang et al., 2019). Moreover, locally unmeasured microsite effects may confound the influence of the spatial distribution on species interaction patterns. In summary, our findings show the role of niche/dispersal-related processes (as implied by spatial overlap) in organizing symbiotic patterns of plant-EM fungal communities. This in turn points to the importance of co-occurrence as regulated by the effects of belowground environmental filtering in generating the observed links between plants and EM fungi.

### Prediction of Network-Aggregated Statistics

Though species abundance makes only a weak contribution to the species interaction frequency of plants and EM fungi, we found that species abundance matching and spatial constraints made the highest contribution to most network metrics (nestedness included, Figures 2A–C,F). The mechanisms underlying the effect of species abundance on network structure (e.g., nestedness) could be explained by the regulation of right-skewed frequency distributions of relative species abundance on co-occurrence patterns (Canard et al., 2014). Moreover, spatial autocorrelation over short distances (in a range of 25 m, see Supplementary Figure S7) and limited dispersal capacity (Tedersoo et al., 2011; Bahram et al., 2013) may lead to aggregated distributions of EM fungi. In addition, the nonrandom distribution of host plants is also dependent to some extent on the occurrence of suitable mycorrhizal fungi (Jacquemyn et al., 2013). This may explain how spatial overlap of plants and fungi can predict network assembly (Jacquemyn et al., 2015).

However, co-occurrences of plant-EM fungi in a microsite were only a prerequisite to establishing symbiotic relationships. For example, the most dominant EM species that co-occur at a given study site are associated with distinct plants with scattered distributions (Tedersoo et al., 2010). This suggests that other biological constraints such as trait-matching of plant-fungal networks might contribute to the observed network structure (i.e., network specificity and modularity, Figures 2D,E). For example, some modules were dominated by *Scleroderma* spp. fungi with long-distance exploration while other modules were dominated by *Russula* spp. fungi with contact hyphal exploration (Supplementary Figure S5 and Zhu et al., unpublished 2022). Plant trait-based host selection behavior in EM fungi or sampling effects associated with species abundance and richness of the plant taxa may contribute to observed network specificity and modularity. Besides this, other deterministic factors such as phenology (Donatti et al., 2011; Morente-López et al., 2018) and a combination of local adaptation and competition (Valverde et al., 2020) can affect network modularity. Observed network specialization is poorly predicted by abundance, spatial overlap and plant traits (Sáyago et al., 2013), consistent with our study (Figure 2D).

Our results suggest that species abundance and spatial distribution can better predict the network structures that are regulated by both neutral processes (e.g., relative species abundance) and niche/dispersal related processes (as implied by spatial overlap), while failing in other network structural properties, including modularity, that may be mostly driven by trait-based niche processes (Donatti et al., 2011; Morente-López et al., 2018).

### Phylogenetic Signal of Species Associations

It has been shown that species interaction patterns of plants and root fungi are mainly constrained by the phylogeny of the host plant, including from the study plot (Toju et al., 2016; Wang et al., 2019) and weakly constrained by fungal phylogeny (Munoz et al., 2012). Similarly, we found that plant-fungal interactions were strongly associated with plant phylogeny (one-tailed Mantel test *r* = −0.086, *p* = 0.025) but weakly associated with fungal phylogeny (one-tailed Mantel test *r* = −0.023, *p* = 0.001). A lack of phylogenetic signal in species degree and interaction strength for fungal hosts (Table 2) may be related to the high vulnerability of fungal hosts and only distant relatedness between the multiple EM fungal partners supported. In contrast, phylogenetic constraints are evident for EM fungi on network characteristics, such as network nestedness (Chen et al., 2017) and compartmentalization (Vacher et al., 2008). However, the phylogenetic conservatism for EM fungi in species degree and interaction strength was only supported by λ statistics but not *K* statistics (Table 2). This suggests *K* statistics might underestimate the phylogenetic signal because it is sensitive to the number of missing plant taxa (Jardim et al., 2021).

Overall, a significant but weak phylogenetic signal on plant-EM fungal symbiotic associations suggests that the contribution of conservative traits on the network structure thus may be limited, while traits unconstrained by phylogenetic history may play important roles in regulating plant-fungal interaction patterns. Additionally, evolutionary history may influence network structure at other scales and analyzing a phylogenetically more diverse root fungal community or a larger network might lead to the consistent detection of a phylogenetic signal.

## Conclusion

Taken together, our study for the first time illustrates that spatial overlap, species abundance and phylogeny can be important in structuring a complex plant-EM fungal network. In particular, we found nestedness was accurately predicted by a combination of species abundance and spatial overlap. However, even the best predictor model used here could not accurately reproduce the observed values of some network metrics (notably network modularity and specificity). This implies that complex plant-fungal networks may be difficult to be synthesized sufficiently while only considering spatial distribution and species abundance. A weak phylogenetic constraint was detected in species interactions of plants and root fungi. This suggests phylogeny and functional traits have the potential to contribute to the species associations of plants and EM fungi. More studies will be required to incorporate a variety of root physical and chemical traits to reveal the underlying processes of plant and fungus symbiotic networks. Thus, improvements of these models would be very helpful to illustrate the plant-fungal network patterns in future.

It is also important to acknowledge that species patterns and network estimates may be affected by limitations of DNA sequence clustering algorithms and root microbial communities may be associated with the taxonomic resolution of ITS sequences. Thus, it would be advisable to investigate microbial communities and construct high-resolution plant–root microbial networks using metagenomics technology in future.

## Data Availability Statement

The interaction matrix of plants and ectomycorrhizal fungi, and taxonomic information of ectomycorrhizal fungi are available at: https://doi.org/10.1111/nph.15786 (see Supplementary Material, Wang et al., 2019). The main R scripts are available at: https://github.com/chunchao11/plant-fungal-network.

## Author Contributions

CZ designed, analyzed and prepared the manuscript with input from DD. YCh, WL, ZW, YCa, YL, and MZ revised the manuscript. All authors contributed to the article and approved the submitted version.

## Funding

This project was funded by the Science and Technology Program of Guizhou Province, China (QKHJC- ZK [2021] 096) and the National Natural Science Foundation of China (32101281 to CZ, 31901105 to MZ).

## Conflict of Interest

The authors declare that the research was conducted in the absence of any commercial or financial relationships that could be construed as a potential conflict of interest.

## Publisher’s Note

All claims expressed in this article are solely those of the authors and do not necessarily represent those of their affiliated organizations, or those of the publisher, the editors and the reviewers. Any product that may be evaluated in this article, or claim that may be made by its manufacturer, is not guaranteed or endorsed by the publisher.
