# Supplementary material for: The Effects of Species Abundance, Spatial Distribution, and Phylogeny on a Plant-Ectomycorrhizal Fungal Network
Source: Front Plant Sci. 2022 May 18;13:784778. doi: 10.3389/fpls.2022.784778 (PMC9158544; doi:10.3389/fpls.2022.784778)
Supplement: Supplementary file 1 [file Data_Sheet_1.docx]

# SUPPLEMENTARY MATERIALS

**Figure S1.** A perspective of a 50 ha forest and tree sampling locations. The x-axis ranges from 0 to 1000 meters, the y-axis ranges from 0 to 500 meters, and the height ranges from 437 to 700 meters in the plot. Root samples are randomly collected from 512 wood plants of 43 plant species (shown in yellow circles) in this forest plot.

**
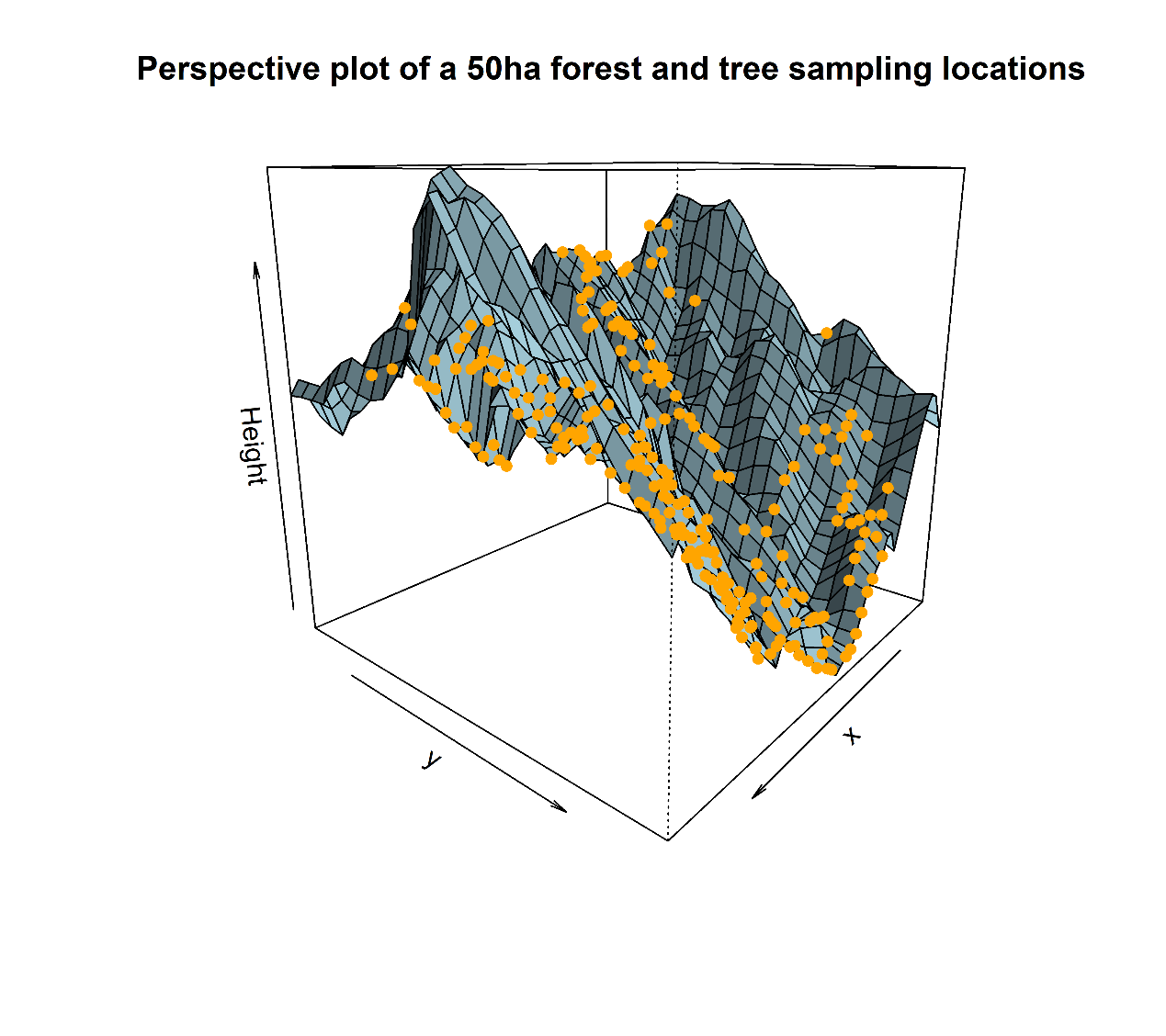
**

**FIGURE S2.** The phylogenetic tree of 43 plant species in a 50-ha subtropical forest plot in the Heishiding nature reserve of Southern China.

**FIGURE S3.** The phylogenetic tree of ectomycorrhizal fungal species in a 50-ha plot in a subtropical forest in the Heishiding Nature Reserve of Southern China. Phylogenetic clades at genus level with no less than 5 species are highlighted using distinct colours with matching genus name labels.

**
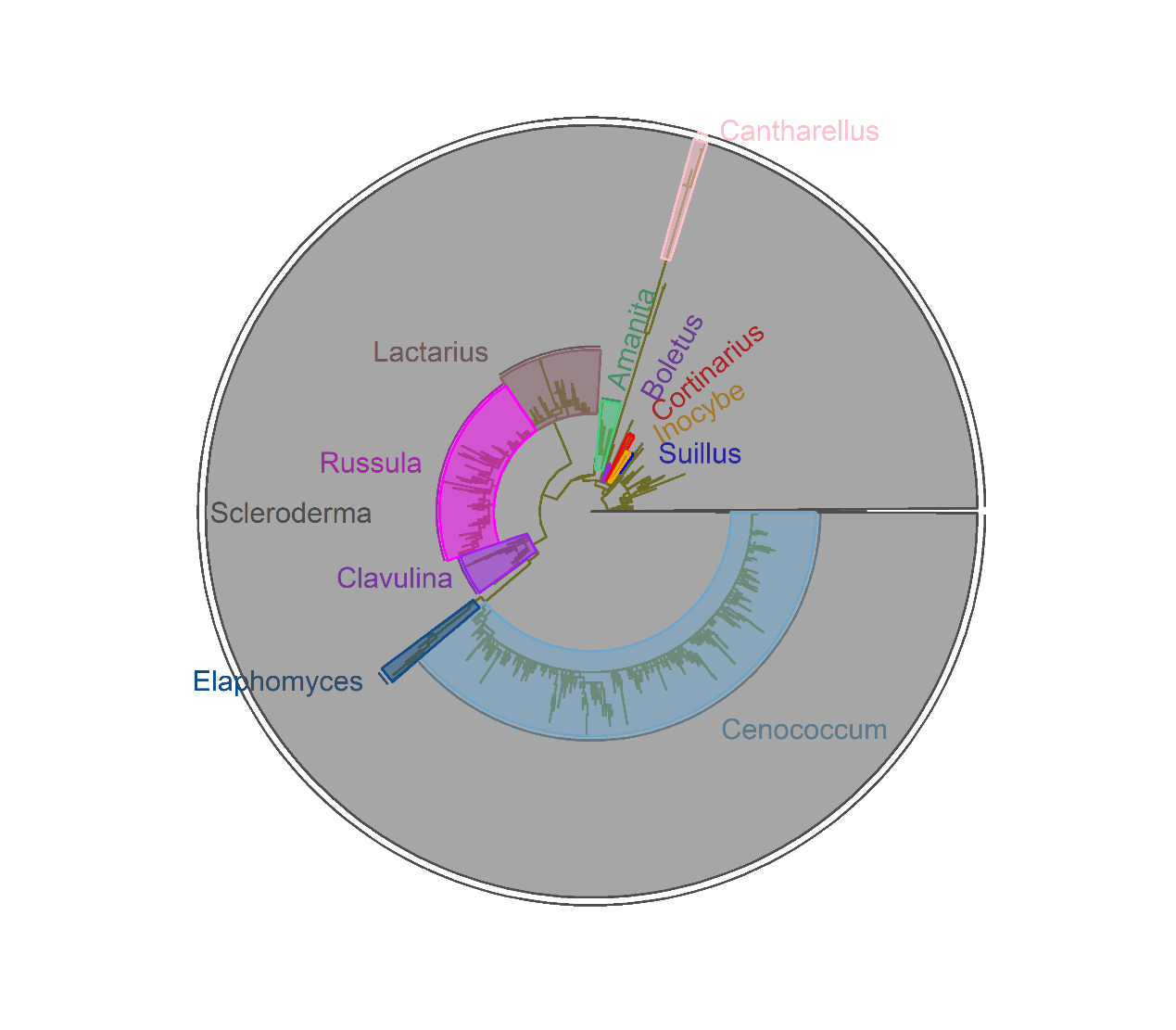
**

**FIGURE S4.** The nested structure of plant-ectomycorrhizal fungal (EM) network. Fungal species are shown in columns and plant species are denoted in rows. Fungal genera are denoted in distinct colours. Log-transformed mean abundance of fungal OTUs sampled from each host tree are filled in each cell on the heatmap. The relative abundance of each fungal species (OTU) on each host tree is evaluated after subsampling each sample to 3000 sequence reads.


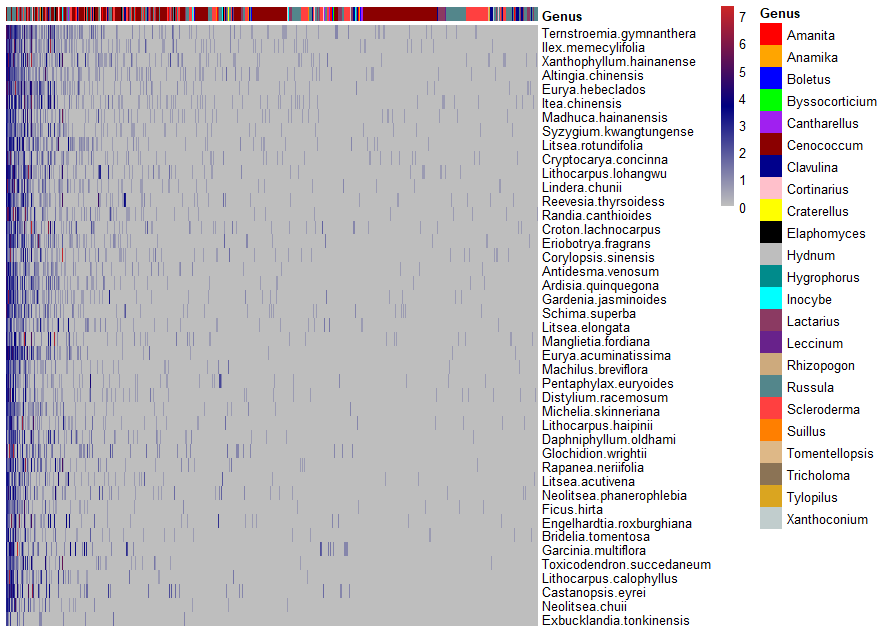


**FIGURE S5.** The modular structure of plant-ectomycorrhizal fungal (EM) network. Plant-EM fungal networks are separated into 14 modules. The modules where plant and EM fungal species are located and fungal genera are shown in distinct colors. Log-transformed mean abundance of fungal OTUs sampled from each host tree are filled in each cell on the heatmap. The abundance of each fungal species (OTU) on each host tree is evaluated after subsampling each sample to 3000 sequence reads.

**
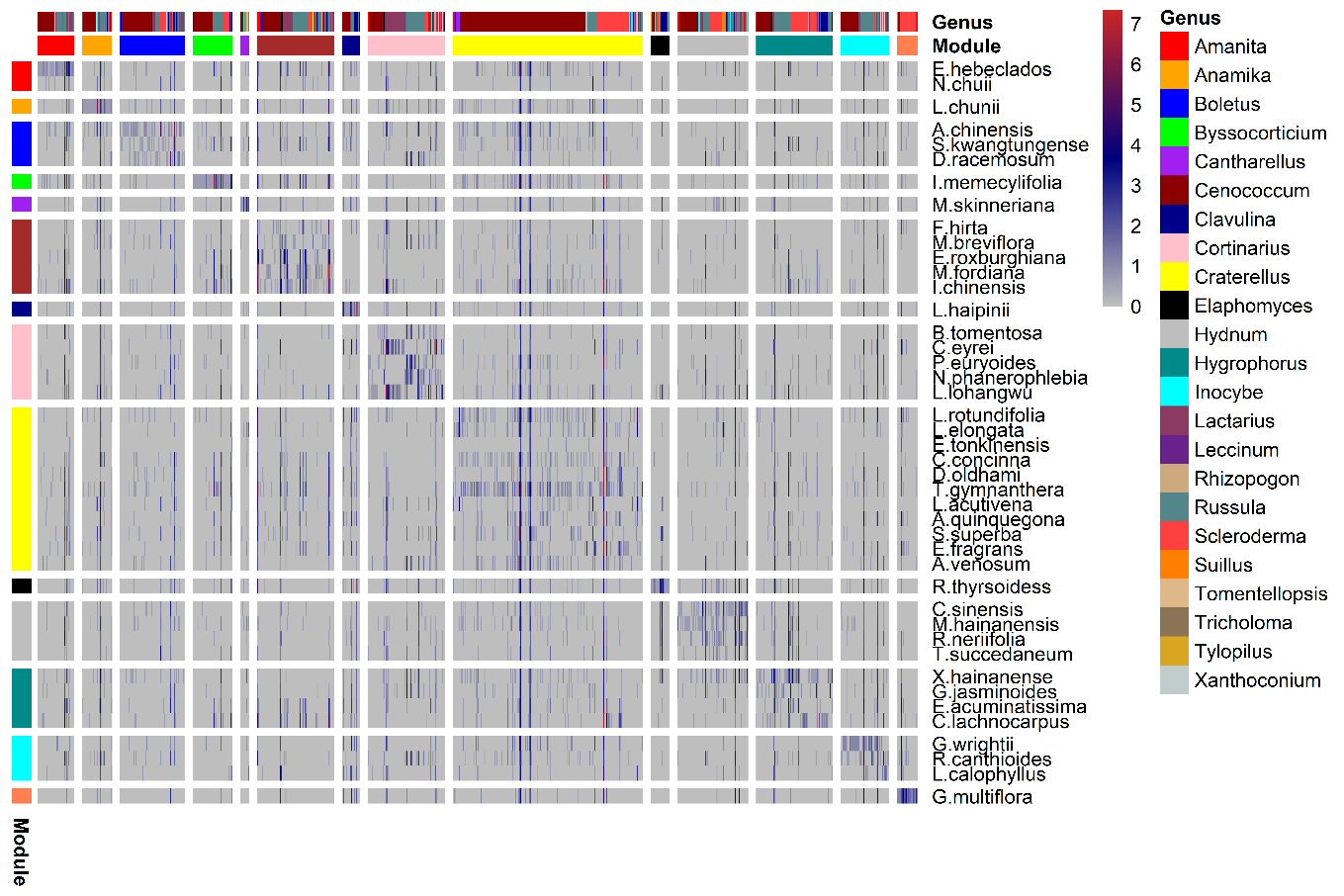
**

**FIGURE S6.** The effects of sampling efforts on network nestedness and modularity. The sampling efforts are evaluated by simulating how network nestedness and modularity vary with an increased number of plant species.

**
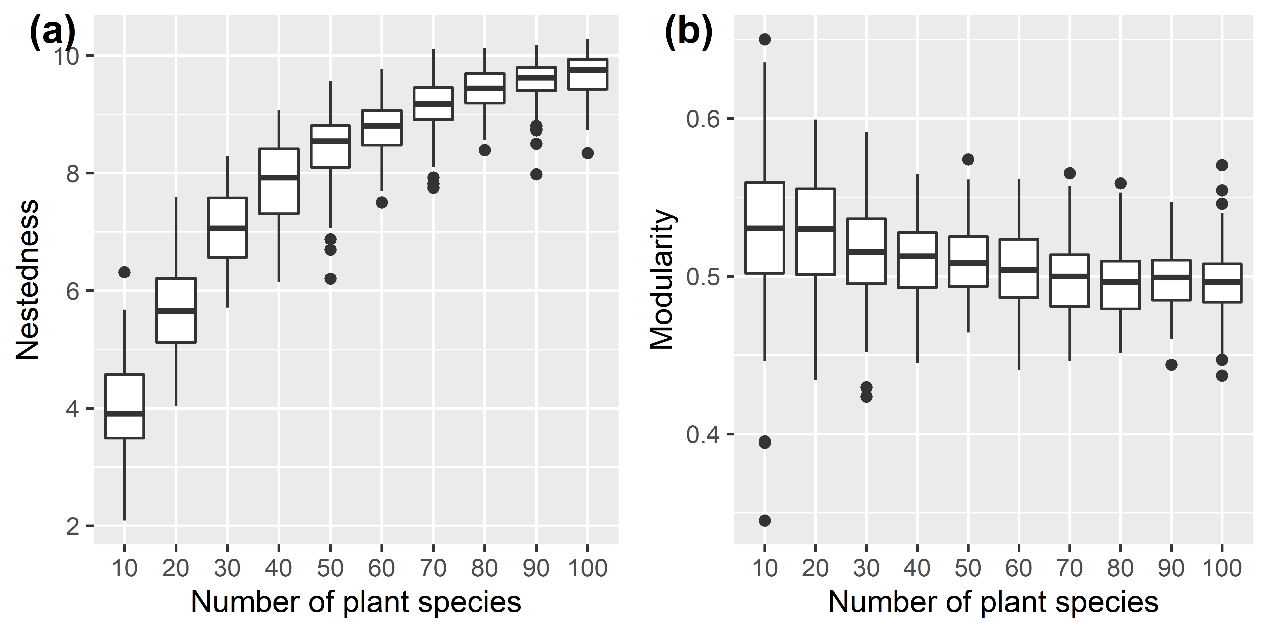
**

**FIGURE S7.** Mantel correlograms of the community composition of plant ectomycorrhizal fungi at different spatial distances. Mantel’s r is calculated from the host spatial distance and fungal community dissimilarity (Hellinger distance) matrices. Solid dots indicate significant values (p-value < 0.05).

**
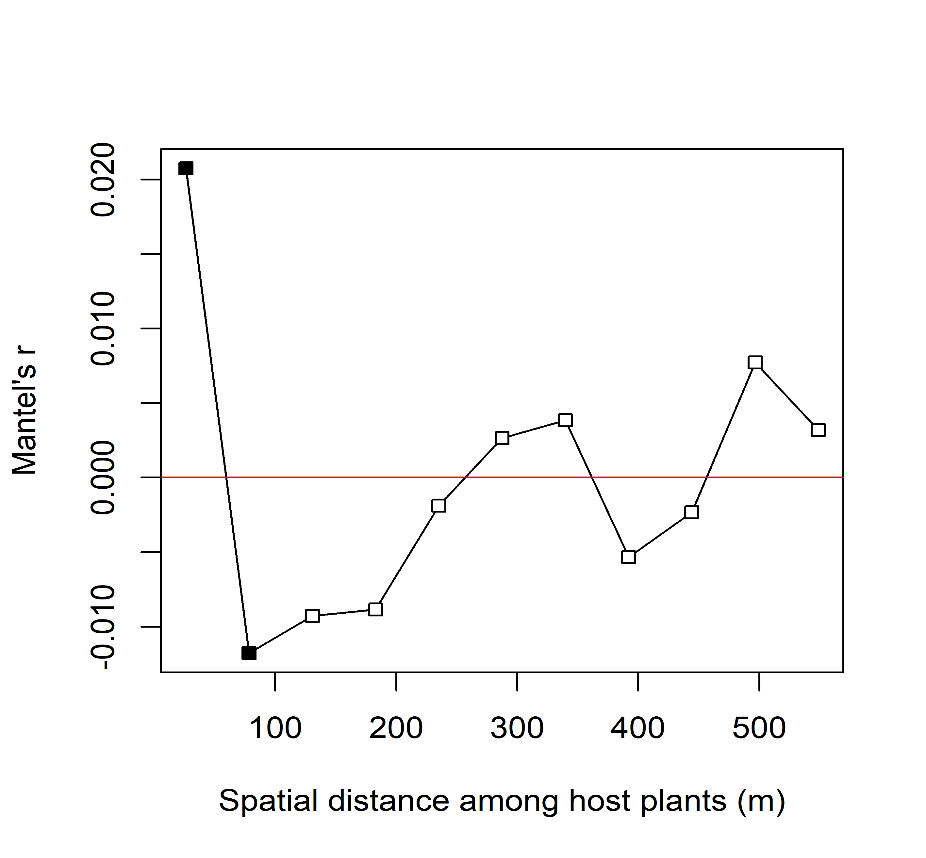
**

**METHOD S1. The phylogenetic construction of ectomycorrhizal fungi (862 species) and host plants (43 species) in a 50 ha subtropical forest plot.**

A phylogenetic tree among host plant species was reconstructed using four general plant DNA barcodes (*rbcLa*, *matK*, *trnL* and ITS2) released after a series of laboratory work (Zhu et al., unpublished). The multiple sequence alignments of host plants aligned in Clustal Omega (<https://www.ebi.ac.uk/Tools/msa/clustalo/>) were used to construct the phylogenetic tree of host plants in RAxML software (Stamatakis, 2014). For the 862 species of ectomycorrhizal fungi, ITS sequences were used to reconstruct the fungal phylogenetic tree (Zhu et al., unpublished). The GUIDANCE2 software (Sela et al., 2015) was used to evaluate the quality scores of multiple sequence alignments of ITS sequences aligned by MAFFT (Katoh and Standley, 2013). Using multiple sequence alignments from MAFFT removing low-quality columns (below 0.93), we constructed the phylogenetic tree of ectomycorrhizal fungi in RAxML software (Stamatakis, 2014). Fungal phylogeny was constructed with a constraint backbone phylogeny, which was generated based on fungal taxonomic ranks using a Perl script (Tedersoo et al., 2018). The maximum likelihood phylogenies for both 862 species of mycorrhizal fungi and 43 species of plants were inferred using the GTR + G model with default settings (Stamatakis, 2014).

**REFERENCES**

Katoh, K., and Standley, D. M. (2013). MAFFT multiple sequence alignment software version 7: Improvements in performance and usability. *Mol. Biol. Evol.* 30, 772–780. doi:10.1093/molbev/mst010.

Sela, I., Ashkenazy, H., Katoh, K., and Pupko, T. (2015). GUIDANCE2: Accurate detection of unreliable alignment regions accounting for the uncertainty of multiple parameters. *Nucleic Acids Res.* 43, W7–W14. doi:10.1093/nar/gkv318.

Stamatakis, A. (2014). RAxML version 8: a tool for phylogenetic analysis and post-analysis of large phylogenies. *Bioinformatics* 30, 1312–1313. doi:10.1093/bioinformatics/btu033.

Tedersoo, L., Sánchez-Ramírez, S., Kõljalg, U., Bahram, M., Döring, M., Schigel, D., et al. (2018). High-level classification of the Fungi and a tool for evolutionary ecological analyses. *Fungal Divers.* 90, 135–159. doi:10.1007/s13225-018-0401-0.
